# Harnessing the potential of LPMO-containing cellulase cocktails poses new demands on processing conditions

**DOI:** 10.1186/s13068-015-0376-y

**Published:** 2015-11-25

**Authors:** Gerdt Müller, Anikó Várnai, Katja Salomon Johansen, Vincent G. H. Eijsink, Svein Jarle Horn

**Affiliations:** Department of Chemistry, Biotechnology and Food Science, Norwegian University of Life Sciences, P. O. Box 5003, 1432 Ås, Norway; Biofuels Technology, Novozymes A/S, Krogshøjvej 36, 2880 Bagsværd, Denmark; Division of Industrial Biotechnology, Chalmers University of Technology, Kemivägen 10, 412 96 Gothenburg, Sweden

**Keywords:** GH61, Biorefinery, Cellulase, AA9, Lignin, Bioeconomy, Biofuel

## Abstract

**Background:**

The emerging bioeconomy depends on improved methods for processing of lignocellulosic biomass to fuels and chemicals. Saccharification of lignocellulose to fermentable sugars is a key step in this regard where enzymatic catalysis plays an important role and is a major cost driver. Traditionally, enzyme cocktails for the conversion of cellulose to fermentable sugars mainly consisted of hydrolytic cellulases. However, the recent discovery of lytic polysaccharide monooxygenases (LPMOs), which cleave cellulose using molecular oxygen and an electron donor, has provided new tools for biomass saccharification.

**Results:**

Current commercial enzyme cocktails contain LPMOs, which, considering the unique properties of these enzymes, may change optimal processing conditions. Here, we show that such modern cellulase cocktails release up to 60 % more glucose from a pretreated lignocellulosic substrate under aerobic conditions compared to anaerobic conditions. This higher yield correlates with the accumulation of oxidized products, which is a signature of LPMO activity. Spiking traditional cellulase cocktails with LPMOs led to increased saccharification yields, but only under aerobic conditions. LPMO activity on pure cellulose depended on the addition of an external electron donor, whereas this was not required for LPMO activity on lignocellulose.

**Conclusions:**

In this study, we demonstrate a direct correlation between saccharification yield and LPMO activity of commercial enzyme cocktails. Importantly, we show that the LPMO contribution to overall efficiency may be large if process conditions are adapted to the key determinants of LPMO activity, namely the presence of electron donors and molecular oxygen. Thus, the advent of LPMOs has a great potential, but requires rethinking of industrial bioprocessing procedures.

**Electronic supplementary material:**

The online version of this article (doi:10.1186/s13068-015-0376-y) contains supplementary material, which is available to authorized users.

## Background

In the coming transition from a fossil-based economy to a biomass-based economy, lignocellulosic biomass will play a key role since it is an abundant renewable feedstock that may be converted to fuels and chemicals. However, due to its recalcitrance, biochemical processing of lignocellulose is challenging. One of the main constitutes of this biomass is cellulose, a polymer of β-1,4-linked glucose, which enzymatically can be converted to fermentable glucose. The traditional view has been that this saccharification step involves three main types of enzymes: endo-active cellulases cleaving glycosidic bonds at random, internal positions in the cellulose chain, exo-active cellulases processively cleaving cellobiose from chain ends, and β-glucosidases for converting cellobiose and small cello-oligosaccharides to glucose [1]. The recent discovery of lytic polysaccharide monooxygenases (LPMOs) [2–6] has revolutionized our view on how cellulose is degraded. In contrast to typical cellulases, which are hydrolytic enzymes, LPMOs are metalloenzymes that degrade cellulose using a mechanism involving molecular oxygen and an electron donor [3, 4]. LPMO-catalyzed cleavage leads to oxidation of one of the carbons in the scissile β-1,4-glycosidic bonds, i.e., oxidation of C1 or C4. Lytic polysaccharide monooxygenases act synergistically with cellulases to improve saccharification yields [6–8], and are present in modern commercial cellulase cocktails [9]. The high efficiency of the most recently developed commercial enzyme cocktails has been an important factor in making cellulosic ethanol a commercial reality [10].

Several fundamental studies have been carried out to characterize LPMO activity, leading to the discovery of LPMOs active on cellulose [3, 5, 6, 11], chitin [12, 13], cellodextrins [14], hemicellulose [15], and starch [16, 17]. However, relatively little has been published on how these enzymes perform in an applied setting. Cannella et al. [9] showed that the improved efficiency of the commercial cellulase cocktail Cellic™ CTEC2, relative to Celluclast, was associated with the production of aldonic acids (i.e., C1-oxidized products). Early studies by Harris et al. [7] indicated that substrate composition could affect LPMO activity, and subsequent studies have elaborated on this [8]. Notably, LPMO activity depends on the presence of oxygen [2], which hence is likely to be an important factor to consider when designing industrial saccharification and fermentation processes.

In the present study, we have investigated the role of LPMOs in enzyme cocktails for the conversion of lignocellulosic biomass at high dry matter (DM) concentration. In particular, we have looked at the effects of process conditions that are not usually considered in the enzymatic conversion of biomass, namely access to oxygen and reducing agents, on overall saccharification efficiency and on the formation of oxidized products. Importantly, we were able to quantify C4-oxidized products by a novel method, thus observing LPMO activity directly in the degradation processes. Finally, we determined the relative amount of LPMO needed to optimize the performance of Celluclast, an early commercial cellulase cocktail containing little LPMO activity. The results underpin the importance of LPMOs in current commercial cellulose cocktails and show that efficient enzymatic saccharification of lignocellulosic biomass depends on designing processing conditions that promote LPMO activity.

## Results

Although it is well established that molecular oxygen and an electron donor are required for the action of LPMOs [2], still little is known about the impact of these requirements on the efficiency of commercial cellulase mixtures [18, 19]. In particular, the potentially crucial role of oxygen has not been addressed in applied settings. Therefore, cellulosic (Avicel) and lignocellulosic [steam-exploded (SE) birch] substrates were subjected to LPMO-containing cellulase cocktails at high substrate loading (10 and 20 % w/w, DM) in the presence or absence of oxygen and an electron donor (ascorbic acid).

**Fig. 1:**
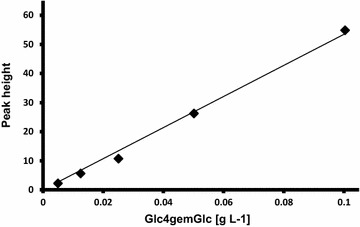
Correlation between HPAEC peak height and concentration of Glc4gemGlc

For quantification of LPMO activity, we developed a method to quantify C4-oxidized products. *Nc*LPMO9C was used to produce Glc4gemGlc (C4-oxidized cellobiose) from cellopentaose, and a linear correlation was found between the concentration of Glc4gemGlc (0.005–0.1 g/L) and high-performance anion exchange chromatography (HPAEC) peak height (see Fig. 1; Additional file 1: Figure S1 and Table S1).

**Fig. 2:**
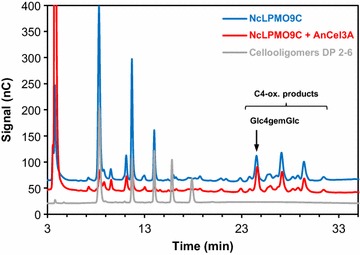
HPAEC chromatogram showing the effect of *An*Cel3A β-glucosidase on the reaction products of *Nc*LPMO9C, a C4-oxidizing LPMO. The *blue curve* shows products (Glc2, Glc3, Glc4, and oxidized sugars) generated from PASC by *Nc*LPMO9C. The *red curve* shows the product profile after *An*Cel3A has been added to the *Nc*LPMO9C product mix. The *grey curve* shows cello-oligosaccharide standards from Glc_2_ to Glc_6_, eluting according to length, with Glc_2_ elution first. It is clearly seen that treatment with *An*Cel3A leads to production of Glc (*red* peak to the *left*) and consumption of the native cello-oligosaccharides. However, the C4-oxidized products were not degraded by *An*Cel3A

**Fig. 3:**
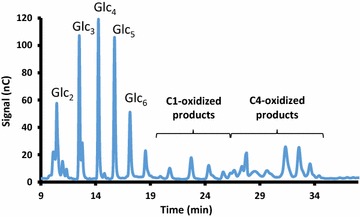
HPAEC chromatogram showing the soluble products obtained after degradation of PASC by *Ta*LPMO9A. The profile indicates a dominating C4-oxidizing activity with minor C1-oxidizing activity

The results below show that Glc4gemGlc accumulated during saccharification of lignocellulosic materials involving LPMO activity, indicating that this dimeric product cannot be cleaved by the β-glucosidase activity in the enzyme cocktail. The inability to cleave Glc4gemGlc was confirmed in a separate experiment where a purified β-glucosidase was added to C4-oxidized products (see Fig. 2). The inability to cleave C4-oxidized sugars is not surprising since the **−**1 subsite of β-glucosidases, where the non-reducing, oxidized sugar moiety would need to bind, is a site with high binding specificity (26). Notably, β-glucosidases do cleave C1-oxidized cello-oligomers to produce gluconic acid [9]. In our experiments, gluconic acid was difficult to quantify due to overlapping peaks in the HPAEC analyses (Additional file 1: Figure S2). Therefore, LPMO activity was monitored by determining the production of Glc4gemGlc, which was the only C4-oxidized sugar accumulating under these conditions and which eluted in a region without overlapping peaks (Additional file 1: Figure S2). In our dosing experiments below, we used a purified LPMO which can oxidize both C1 and C4, but which primarily oxidizes C4 (see Fig. 3).

### Saccharification of cellulose

**Fig. 4:**
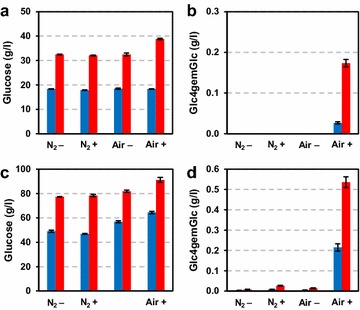
Saccharification of Avicel at 10 % (**a**, **b**) or 20 % (**c**, **d**) DM at aerobic (“Air”) or anaerobic (“N_2_”) conditions in the presence (“+”) or absence (“−”) of ascorbic acid. The *left panels* (**a**, **c**) show glucose concentrations at two time points, while the *right panels* (**b**, **d**) show production of Glc4gemGlc at the same time points. For panels **a** and **b**, *blue bars* represent 4 h of incubation, while *red bars* represent 18 h. For panels **c** and **d**, *blue bars* represent 18 h of incubation, while *red bars* represent 46 h

First, Avicel (microcrystalline cellulose) was treated at 10 and 20 % (w/w) DM with the LPMO-containing cellulose cocktail Cellic™ CTEC2 under aerobic and anaerobic conditions in the absence or presence of ascorbic acid (Fig. 4). For the reactions at 20 % (w/w) cellulose, longer incubation periods were used (46 vs 18 h for the 10 % reactions) since saccharification efficiency is decreased at such high DM concentration [20]. At both substrate concentrations, the highest glucose yields were obtained in reaction mixtures containing both air and an added electron donor (Fig. 4a, c). Omitting either molecular oxygen (in the air) or ascorbic acid, or both led to equal decreases in glucose yields. The higher glucose yields in the reactions with air and reducing agent were positively correlated with the release of C4-oxidized sugars (Fig. 4b, d), which were estimated to constitute 0.5 and 0.6 % of the total released sugars in the 10 and 20 % reactions, respectively. In the absence of oxygen or ascorbic acid, Cellic™ CTEC2 produced hardly any oxidized sugars from Avicel, as one would expect if there were little LPMO activity during the reaction. Interestingly, for both the 10 and 20 % DM reactions, the relative amount of oxidized sugars (as compared to glucose) was higher at the end of the reaction than earlier in the reaction.

### Saccharification of steam exploded birch

**Fig. 5:**
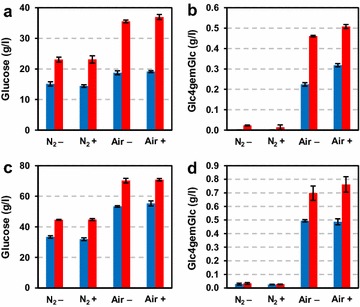
Saccharification of SE birch wood at 10 % (**a**, **b**) or 20 % (**c**, **d**) DM at aerobic (“Air”) or anaerobic (“N_2_”) conditions in the presence (“+”) or absence (“−”) of ascorbic acid. The *left panels *(**a**, **c**) show the glucose concentration at different time points, while the* right panels* (**b**, **d**) show production of Glc4gemGlc. The color-coding and the incubation times are as in Fig. 4

The experiments carried out for cellulose were repeated with SE birch wood, representing a relevant industrial substrate (Fig. 5). The conditions used for steam explosion pretreatment of birch (210 °C for 10 min) were selected based on a previous study where pretreatment was optimized for maximizing enzymatic saccharification yield [21]. Strikingly, at both 10 and 20 % (w/w) DM loading aerobic conditions led to the release of about 60 % more glucose compared to anaerobic conditions (Fig. 5a, c). The higher glucose yield in the presence of oxygen was associated with release of C4-oxidized sugars (Fig. 5b, d). In contrast to Avicel saccharification, the addition of a reducing agent had no effect on the saccharification yield. The relative amounts of oxidized sugars for SE birch were estimated to constitute 1.2–1.6 % of the total amount of released sugars, that is, more than double the amount released during depolymerization of Avicel. Unlike the reactions with Avicel, no relative accumulation of oxidized products in the later stage of the reaction was observed.

**Fig. 6:**
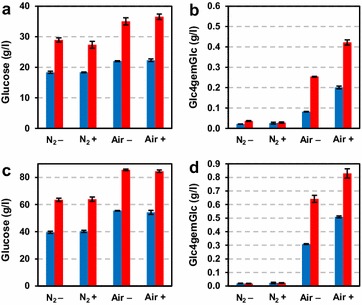
Saccharification of washed SE birch wood at 10 % (**a**,** b**) or 20 % (**c**, **d**) DM at aerobic (“Air”) or anaerobic (“N_2_”) conditions in the presence (“+”) or absence (“−”) of ascorbic acid. The *left panels* (**a**, **c**) show the glucose concentration at different time points, while the *right panels* (**b**, **d**) show production of Glc4gemGlc. The color-coding and the incubation times are as in Fig. 4

To investigate whether soluble compounds in SE birch could play the role of electron donor, the experiment was repeated with extensively washed SE birch at both 10 and 20 % (w/w) DM (Fig. 6). In the reactions carried out under anaerobic conditions, glucose release from the washed SE birch substrate (Fig. 6a, c) exceeded that from the unwashed SE birch (Fig. 5a, c) at both sampling points, meaning faster saccharification and higher final conversion yields. The yield increase amounted to approximately 20 and 40 % at 10 and 20 % (w/w) DM, respectively. This indicates that some of the water-soluble compounds present in SE birch inhibit one or more of the enzymes in Cellic™ CTEC2. As expected, addition of ascorbic acid had no effect under these anaerobic conditions.

Washing also led to higher yields when applying aerobic conditions, albeit to a lesser extent than under anaerobic conditions, and only for the high DM reaction, where the increase in final yield was approximately 20 % (Figs. 6c vs  5c). Somewhat unexpectedly, the saccharification yield was still independent of the addition of an external electron donor (Fig. 6a, c). However, the amount of oxidized sugars did increase in the presence of ascorbic acid (Fig. 6b, d). Taken together, these results show that the washed SE birch still contains a reducing potential that is accessible to LPMOs, but that the electron-donating capacity has been reduced by the washing. The fact that the overall saccharification by Cellic™ CTEC2 was not negatively affected by washing the substrate is likely to reflect the beneficial effect of removing enzyme inhibitors, as observed under anaerobic conditions.

### LPMO dose

To investigate the optimal amount of LPMOs in a cellulase preparation, we used a blend of Celluclast and Novozym 188, which is considered an LPMO-free cellulase preparation.

**Fig. 7:**
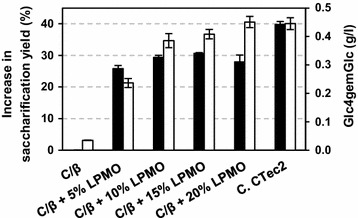
Increase in saccharification yield and production of oxidized sugars upon supplying Celluclast with *Ta*LPMO9A. Steam-exploded birch at 10 % DM was incubated with a Celluclast/Novozym 188 mixture (“C/β”) for 20 h. Various fractions (0–20 %) of the enzyme cocktail were replaced by the LPMO. *Black bars* show the increase in glucose release (in %) compared to the Celluclast/Novozym 188 mixture alone, whereas the *white bars* show the concentration of oxidized sugars in the product mixtures. For comparison, the results for a reaction with Cellic™ CTEC2 (“C. CTec2”) are included

**Fig. 8:**
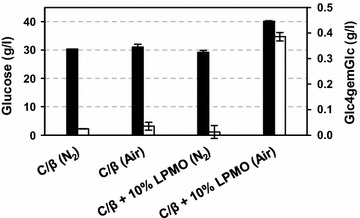
The effect of oxygen on mixtures of Celluclast and *Ta*LPMO9A. Reaction conditions were as in Fig. 4. Production of glucose is indicated by the *black bars*, and production of Glc4gemGlc is indicated by the *white bars*. Reactions were run under aerobic (“Air”) or anaerobic (“N_2_”) conditions, and the enzyme mixtures used were the Celluclast/Novozym 188 (C/β) blend and the same blend but with 10 % of the protein substituted by *Ta*LPMO9A

Celluclast is a cellulase cocktail produced by *Trichoderma reesei*, which is known to harbor three LPMO genes in its genome. However, none of these LPMOs are expressed at high levels [7, 22]. The Celluclast/Novozym 188 mixture was partially replaced with different amounts of a purified LPMO from *Thermoascus aurantiacus* (Fig. 3), substituting between 5 and 20 % of the protein in the mixture (Fig. 7). Maximum glucose yields from SE birch, which were 31 % higher compared to the reaction without LPMO, were obtained by replacing 15 % of the protein in the Celluclast/Novozym 188 blend with *Ta*LPMO9A. The amount of oxidized sugars released correlated with the amount of added LPMOs, and at 20 % added LPMO this amount was similar to that obtained in the reaction Cellic™ CTEC2. Notably, glucose yields were below those obtained with Cellic™ CTEC2 in all reactions with Celluclast/Novozym 188/*Ta*LPMO9A blends (Fig. 7). Glucose release by the Celluclast/Novozym 188 mixture was not affected by the presence of air, whereas increased saccharification upon addition of *Ta*LPMO9A only occurred under aerobic conditions (Fig. 8). This latter observation underpins the crucial role of oxygen for efficient LPMO-aided biomass saccharification.

## Discussion

In this study, we have investigated the effects of the presence of air (oxygen) and a reductant (ascorbic acid) on the efficiency of LPMO-containing cellulase preparations in biomass saccharification. We used a relatively pure cellulosic substrate, Avicel, and two more realistic lignocellulosic substrates, unwashed and washed SE birch, at industrially relevant DM concentrations.

Our experiments with the model cellulose substrate Avicel (Fig. 4) unambiguously show that both oxygen and an electron donor are necessary to stimulate LPMO activity in commercial enzyme cocktails and that such stimulation leads to increased saccharification yields. We were able to monitor actual LPMO activity using a newly developed method for detecting oxidized products, and this showed that the generation of oxidized sugars, i.e., LPMO activity, correlated positively with saccharification yield. The experiments with SE birch showed more clearly that keeping aerobic conditions is essential to take full advantage of the potential of modern LPMO-containing cellulase cocktails. The observed 60 % increase in saccharification yield will have considerable economic effects in commercial applications. Obviously, increased yields may be translated into lower feedstock costs. The higher saccharification rate also observed under aerobic conditions could mean shorter processing times or lower overall enzyme loadings.

The data depicted in Figs. 5 and 6 show that for a lignocellulosic substrate, addition of an external electron donor may not be necessary, especially if the substrate is not washed after pretreatment. Realistic lignocellulosic substrates such as SE birch contain, in addition to cellulose, also hemicellulose (xylan) and lignin (Table 1). Lignin is known to take part in redox cycles [23], and hence it is likely that it may act as an electron donor for LPMOs. The production of oxidized sugars was somewhat retarded in the reactions with washed SE birch compared to unwashed SE birch, indicating a role of soluble compounds in boosting LPMO activity. Nevertheless, the remaining solid material alone was capable of supplying electrons even in the washed substrate.

**Table 1:** Carbohydrate and lignin content of substrates (in %)

Substrate	Arabinan	Galactan	Glucan	Xylan	Lignin
SE birch	0.3 ± 0.0	0.9 ± 0.0	43.9 ± 1.1	10.4 ± 0.3	36.5 ± 0.3
Washed SE birch	0.0 ± 0.0	0.1 ± 0.0	49.0 ± 1.6	4.0 ± 0.1	40.7 ± 0.1
Avicel	0.0 ± 0.0	0.0 ± 0.0	92.2 ± 1.7	2.1 ± 0.0	0.9 ± 0.0

Generally, the presence of lignin is regarded disadvantageous in enzymatic saccharification because it forms a physical barrier for the cellulases and because cellulases tend to bind unproductively to this polyaromatic material [24]. However, the new generation of enzyme cocktails with LPMOs requires an electron donor, which is a role lignin can play. It has been pointed out that the ability of lignin to activate LPMOs is dependent on the type of pretreatment applied [25]. Thus, the beneficial effect of lignin on LPMO activity is a new factor that needs to be considered when choosing and developing pretreatment strategies for lignocellulosic biomass. Interestingly, prior to the discovery of LPMOs, Harris et al. observed that the boosting effect of a GH61 protein (now known to be an LPMO) on cellulase activity only occurred on lignocellulosic substrates but not on a pure cellulose substrate, leading to the suggestion that lignin (or hemicellulose) could play a role in this phenomenon [7].

Saccharification of washed SE birch under anaerobic conditions, i.e., with no LPMO activity, gave higher yields compared to saccharification of unwashed SE birch. This is presumably due to the removal of inhibitory compounds originating from the steam explosion pretreatment, which is a process known to potentially generate cellulase inhibitors such as furfurals [26]. This is supported by the fact that the effect of washing was the most prominent at the higher (20 % DM) substrate concentration where the concentration of inhibitors is the highest. It is interesting to note that the effect of washing was bigger under anaerobic conditions, where the LPMOs are not active. There are several possible explanations for this observation, one of the more straightforward ones being that, under aerobic conditions, the presence of LPMO activity somehow compensates for reduced activity of one or more of the cellulases in Cellic™ CTEC2.

Our data indicate that the optimal amount of added *Ta*LPMO9A in a Celluclast background is in the order of 15 %. Despite this high fraction of LPMO, the amounts of oxidized sugars produced were low compared to the amount of glucose. This could be explained by the fact that LPMOs seem to be slow enzymes, with apparent rates that are one–two orders of magnitude lower than the rates of, e.g., cellobiohydrolases [2, 15]. Notably, very little is known about LPMO kinetics and the rate-limiting step in the LPMO reaction, and further studies on the kinetics of these important enzymes are much needed. It is noteworthy that limiting the production of oxidized sugars may be beneficial because this represents an energy loss, the size of which depends on the ability of the organism to ferment oxidized sugars.

This study is the first to report production of C4-oxidized sugars by commercial cellulase preparations during saccharification of a lignocellulosic substrate. Previously, a handful of studies have described production of C1-oxidized sugars in applied settings [9, 18, 25]. These studies were carried out using Cellic™ CTEC2 on pretreated and washed agricultural residues (corn stover, bagasse, and wheat straw). In this study, we aimed at quantifying both types of oxidized sugars; however, due to reasons described above, we were unable to quantify C1-oxidized sugars. Our study complements these earlier studies and shows that Cellic™ CTEC2 produces both types of oxidized sugars. While the total amount of oxidized sugars reported above is underestimated, as C1-oxidized sugars are not accounted for, the correlations between the presence of LPMOs and/or reductants and/or oxygen and saccharification yields are evident and lead to clear conclusions. It has been reported previously that both the type of biomass and the type of pretreatment used affect the level of sugar oxidation [25]. Hydrothermal pretreatment, which was also used in the present study, has been reported to lead to the highest yields of C1-oxidized products, accounting for up to 0.8 % of sugars released from wheat straw [25]. This level is similar to the levels of C4-oxidized sugars observed in this study.

In 1950, Reese et al. [27] presented the C_1_–C_*x*_ theory which implies that efficient hydrolytic conversion of cellulose by cellulases (C_*x*_) requires some sort of enzymatic activation step, catalyzed by C_1_, that makes the crystalline material more accessible. Interestingly, in 1974, while studying saccharification of cellulose by culture filtrates of white-rot fungi, Eriksson and co-workers [28] observed that cellulose degradation was more efficient under aerobic conditions compared to anaerobic conditions. One hypothesis then was that the C_1_ step in Reese’s model involved an oxidative enzyme. The discovery of LPMOs [2], accumulating knowledge on how these enzymes interact with the substrate [29] and the data presented above, explains the observations by Eriksson et al. and is compatible with the C_1_–C_*x*_ theory. One reason that it took some 35 years to discover the LPMOs and explain Eriksson’s observations is probably that the main model organism used in cellulose degradation studies has been *T. reesei*, a fungus with low LPMO activity.

## Conclusions

In this study, we demonstrate a direct correlation between saccharification yield and LPMO activity of commercial enzyme cocktails. Importantly, we show that the LPMO contribution to overall saccharification efficiency may be large if process conditions are adapted to the key determinants of LPMO activity, namely the presence of electron donors and molecular oxygen. Thus, the inclusion of LPMOs in commercial enzyme cocktails has a great potential, but requires rethinking of industrial bioprocessing procedures.

## Methods

### Cellulose substrates and pretreatment

Avicel^®^ PH 101 (Sigma Aldrich, St. Louis, USA) and milled birch wood (*Betula pubescens*) SE at 210 °C for 10 min [21] were used as substrates. Additionally, a sample of SE birch was extensively washed by six cycles of resuspending in Milli-Q water followed by centrifugation at 8000 rpm for 15 min, in order to prepare a substrate without the soluble compounds generated during pretreatment. Phosphoric acid swollen cellulose (PASC), used for characterization of LPMO activity, was prepared from Avicel using the method described by Wood [30].

### Enzymes

Purified AA9 LPMO from *T. aurantiacus* (*Ta*GH61A/*Ta*LPMO9A), β-glucosidase (Novozym 188), and the cellulase cocktails Celluclast 1.5 L and Cellic™ CTEC2 were provided by Novozymes A/S (Bagsvaerd, Denmark). Another AA9 LPMO from *Neurospora crassa* (*Nc*LPMO9C), used for producing C4-oxidized standard, was expressed recombinantly in *Pichia pastoris* clone X-33 [31] under the AOX1 promoter and purified in two steps. After buffer exchange to 50 mM Na–acetate pH 5.0, 50 mL culture broth was loaded onto three 5-mL HiTrap SP HP columns coupled in series (GE Healthcare Life Sciences) at 5 mL/min. Unbound proteins were washed off with 50 mM Na acetate pH 4.0, after which a 0–0.7 M NaCl gradient was applied over 150 mL to elute bound proteins. The peak that eluted at 0.30–0.65 M NaCl was pooled and mixed with a saturated (NH_4_)_2_SO_4_ solution. The resulting sample, containing 35 % (v/v) saturated (NH_4_)_2_SO_4_ solution, was loaded onto three 5-mL Phenyl HP columns (GE Healthcare Life Sciences) coupled in series and equilibrated with a 65:35 % v/v mixture of 50 mM acetate buffer pH 5.7 and a saturated (NH_4_)_2_SO_4_ solution. After washing off unbound proteins, the fraction of the saturated (NH_4_)_2_SO_4_ solution was linearly decreased from 35 to 0 % over 250 mL. The *Nc*LPMO9C containing fractions were pooled and concentrated using Vivaspin ultrafiltration spin columns with simultaneous exchange of the buffer to 20 mM Tris pH 8.0.

### Saccharification

Saccharification of Avicel and birch substrates was carried out in 50-ml rubber sealed glass bottles (Wheaton, Millville, USA) with a working volume of 10 mL. Anaerobic conditions were obtained by flushing the head space of bottles containing the substrate–buffer suspension with pure nitrogen (YARA, Trondheim, Norway) for 3 min, followed by supplementing with l-cysteine hydrochloride monohydrate (Sigma Aldrich, St. Louis, USA) to a final concentration of 0.025 % (w/v) to remove residual oxygen. Reactions were started by injecting enzymes through the septum. The saccharification reactions were carried out in 50 mM sodium acetate buffer pH 5.0 at 50 °C. For reactions involving birch, the pH was adjusted by adding 0.66 ml of 1 M NaOH per g DM. Avicel and birch substrates with a solid loading of 10 and 20 % (w/w) DM were hydrolyzed with Cellic™ CTEC2 (5 mg protein/g DM) in the presence or absence of 1 mM ascorbic acid. Additionally, SE birch with a solid loading of 10 % (w/w) was hydrolyzed with a mixture of Celluclast and Novozym 188 (5:1 ratio; v/v), where various amounts of protein (determined by the Bradford method) in the mixture were replaced by an equal amount of *Ta*GH61A, maintaining the total enzyme load constant at 5 mg/g. The reaction bottles were mixed using a programmable rotator mixer (Multi RS-60, Montebello Diagnostics A/S, Oslo, Norway) by inverting samples at 38 rpm, causing so-called “free fall” mixing [32]. To avoid sampling errors introduced by the high DM content, the entire reaction mixtures were diluted fourfold in Milli-Q water before samples for analysis were collected, as suggested by Kristensen et al. [20]. This implies that there were separate bottles for each time point. Reactions were stopped by heat inactivation of enzymes at 100 °C for 15 min. The supernatants were collected after centrifugation at 4 °C and 4000 rpm for 15 min (Multicentrifuge X1R, Thermo Scientific, Waltham, USA) and filtered using 96-well plates equipped with 0.45-µm-pore size filter (Merck Millipore Ltd., Tullagreen Carrigtwohill, Ireland) prior to HPLC analysis.

### Compositional analysis

The amounts of structural carbohydrates and lignin in the cellulose and birch substrates were determined according to a standard laboratory analytical procedure developed by the National Renewable Energy Laboratory (NREL/TP-510-42618). Monosaccharides were analyzed by high-performance liquid chromatography (HPLC), as described below.

### Preparation of a Glc4gemGlc HPAEC standard

To quantify the formation of C4-oxidized products during biomass degradation, standards were prepared by treating cello-1,4-β-d-pentaose (Megazyme International, Ireland) with the C4-oxidizing LPMO *Nc*LPMO9C, yielding equimolar amounts of native 1,4-β-d-cellotriose and 4-hydroxy-β-d-xylo-Hex*p*-(1 → 4)-β-d-Glc*p* (Glc4gemGlc). The reaction mixture (1 ml) consisted of 2.5 mg cello-1,4-β-d-pentaose and 56 µg *Nc*LPMO9C in 5 mM Tris pH 8.0 containing 2 mM ascorbic acid. The reaction was carried out at 800 rpm and 33 °C for 24 h in an Eppendorf Thermo mixer, and was stopped by boiling for 10 min. A control reaction without ascorbic acid was run to verify that the *Nc*LPMO9C preparation was free of contaminating background endo-β-1,4-glucanase activity. The amount of Glc4gemGlc generated in the reaction was estimated by quantifying the amount of the other cleavage product, cellotriose, using HPAEC (HPAEC; see below; see also Additional file 1: Figure S1 and Table S1). The product mixture of cellotriose and Glc4gemGlc was used as an external standard to quantify the amount of Glc4gemGlc formed in the saccharification experiments.

### *An*Cel3A β-glucosidase

The effect of *An*Cel3A β-glucosidase on the reaction products of *Nc*LPMO9C was tested in the following way: In 200 µl reaction mixture, 100 µl 1 % PASC was incubated with 25 µg LPMO in 0.1 M Na–citrate buffer pH 6.0 for 20 h at 50 °C in the presence of 1.5 mM ascorbic acid. Then 10 µL sample was taken for HPAEC analysis. The remaining reaction mixture was centrifuged and the supernatant added 75 µg *An*Cel3A (purified from Novozym 188 as previously described; [33]) and incubated for 20 h at 50 °C. Identification of C4-oxidized products was based on previous work with *Nc*LPMO9C [14].

### *Ta*LPMO9A activity

The product profile of *Ta*LPMO9A was investigated in the following way: In 200 µl reaction mixture, 100 µl 0.2 % PASC was incubated with 18 µg LPMO in 0.1 M Na–acetate buffer pH 5.0 for 20 h at 50 °C in the presence of 1.5 mM ascorbic acid. Identification of C4-oxidized products was based on previous work with NcLPMO9C [14].

### HPLC analysis of sugars and oligosaccharides

Monosaccharides (d-glucose, d-xylose) from saccharification experiments were analyzed by High-Performance Liquid Chromatography (HPLC) using a Dionex Ultimate 3000 system (Dionex, Sunnyvale, CA, USA) equipped with a refractive index (RI) detector 101 (Shodex, Japan). Separation was performed using a Rezex ROA-organic acid H^+^(8 %), 300 × 7.8 mm (Phenomenex, Torrance, CA, USA) analytical column, which was operated at 65 °C with 5 mM H_2_SO_4_ as an eluent at a flow rate of 0.6 ml/min. Monosaccharides (d-glucose, d-xylose, l-arabinose, d-mannose, and d-galactose) from compositional analysis, as well as native oligosaccharides (DP2, DP3, DP5) and oxidized sugars (Glc4gemGlc and d-gluconic acid) from enzymatic reactions, were analyzed by HPAEC using a Dionex ICS 3000 system (Dionex, Sunnivale, CA, USA) equipped with a CarboPac PA1 column operated at 30 °C, and with pulsed amperometric detection (PAD). Monosaccharides were eluted isocratically at a flow rate of 0.25 ml/min using 1 mM KOH, generated with eluent generator. Native oligosaccharides and oxidized sugars (4-ketoaldose) were separated by applying a gradient with increasing concentration of sodium acetate over time as described by Westereng et al. [34].

The data were collected and analyzed using Chromeleon 7.0.

## Additional file

10.1186/s13068-015-0376-y Degradation of Glc5 by *Nc*LPMO9C from *N. crassa*, **Figure S2.** HPAEC-PAD profiles of saccharification products, **Table S1.** Quantification of oligosaccharide peaks shown in Figure S1.

## Abbreviations

Glc4gemGlc: 4-hydroxy-β-d-xylo-hexopyranosyl-(1 → 4)-β-d-glucopyranosyl
HPLC: high-performance liquid chromatography
HPAEC: high-performance anion exchange chromatography
LPMO: lytic polysaccharide monooxygenase
PASC: phosphoric acid swollen cellulose
SE: steam exploded
