# Supplementary material for: Harnessing the potential of LPMO-containing cellulase cocktails poses new demands on processing conditions
Source: Biotechnol Biofuels. 2015 Nov 25;8:187. doi: 10.1186/s13068-015-0376-y (PMC4659242; doi:10.1186/s13068-015-0376-y)
Supplement: Supplementary file 1 — 10.1186/s13068-015-0376-y Degradation of Glc5 by NcLPMO9C from N. crassa, Figure S2. HPAEC-PAD profiles of saccharification products, Table S1. Quantification of oligosaccharide peaks shown in Figure S1. [file 13068_2015_376_MOESM1_ESM.pdf]

Supplementary Table and Figures

Harnessing the potential of LPMO-containing cellulase cocktails poses new demands on processing conditions

Gerdt Müller[1)], Anikó Várnai[1)], Katja Salomon Johansen[2, 3)], Vincent G.H. Eijsink[1)], Svein Jarle Horn[1)]*

[1)] Department of Chemistry, Biotechnology and Food Science, Norwegian University of Life Sciences, P. O. Box 5003, N-1432 Ås, Norway

[2)] Biofuels Technology, Novozymes A/S, Krogshøjvej 36, DK-2880 Bagsværd, Denmark

[3)] Division of Industrial Biotechnology, Chalmers University of Technology, Kemivägen 10, SE-412 96 Göteborg, Sweden

*Corresponding author, E-mail: svein.horn@nmbu.no

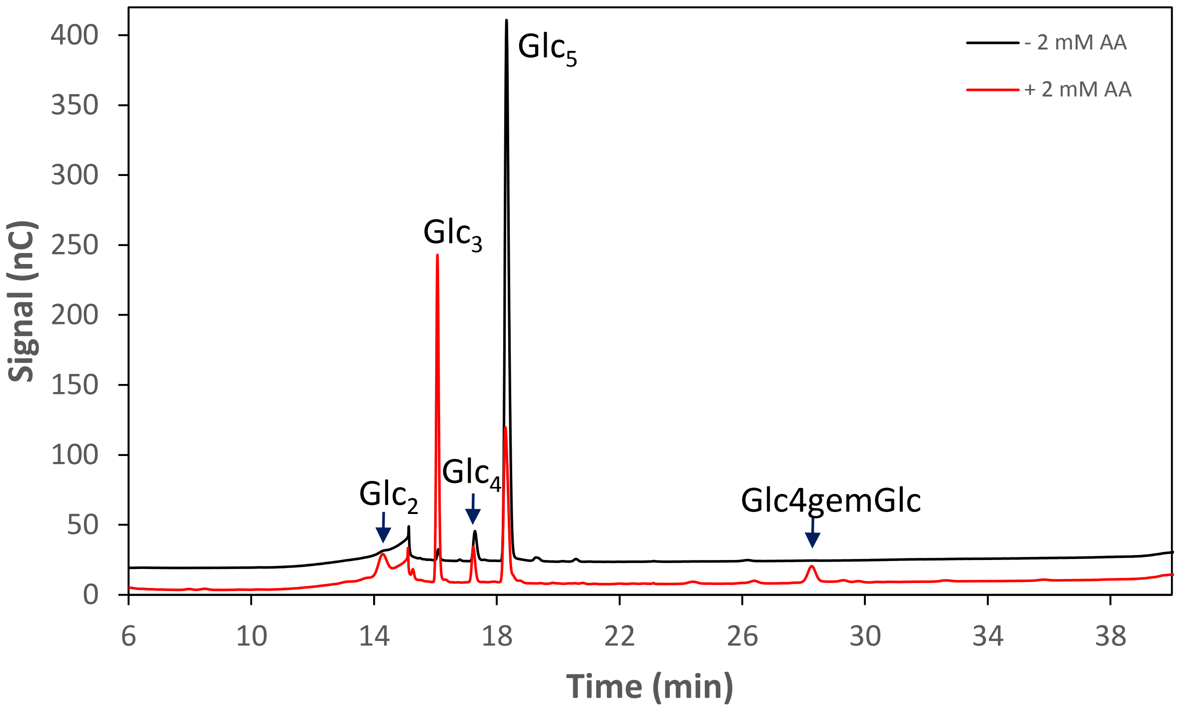

Figure S1. Degradation of Glc_5 by *Nc*LPMO9C from *N. crassa*. Incubation with (red line) and without (black line) ascorbic acid (AA). Incubation of the substrate without enzyme (not shown) yielded the same chromatogram as incubation with enzyme but without AA; note that the Glc_5 preparation contained some Glc_4 impurities. The data show that Glc_5 is mainly converted to Glc_3 and Glc4gemGlc. Reaction conditions were as described in the Materials and methods section. In short: 2.5 mg Glc_5 was incubated with 56 µg *Nc*LPMO9C in 5 mM Tris pH 8.0 at 33 °C for 24 h with or without 2 mM ascorbic acid.

Table S1: Quantification of oligosaccharide peaks shown in Figure S1. Concentrations are given in mM.

	Glc_2	Glc_3	Glc_4	Glc_5	Glc4gemGlc	$Glc4gemGlc_2$
Glc_5 + LPMO - AA	0.00	0.04	0.20	3.03	0.00	0.00
Glc_5 + LPMO + AA	0.34	1.65	0.20	0.99	1.65	0.34
Difference	0.34	1.61	0.00	-2.04	1.65	0.34

Native cello-oligosaccharides were quantified with standards for Glc_2 to Glc_5.The amount of C4-oxidized cello-oligosaccharides, Glc4gemGlc and $Glc4gemGlc_2$, was estimated by assuming equimolar production of Glc4gemGlc and Glc_3, as well as $Glc4gemGlc_2$ and Glc_2. The total consumption of Glc_5 (2.04 mM) was close to the sum of Glc_2 and Glc_3 (1.94 mM). The reaction mixtures (1 ml) consisted of 2.5 mg Glc_5 (3.0 mM) and 56 µg *Nc*LPMO9C in 5 mM Tris pH 8.0 with or without 2 mM ascorbic acid. The reactions were carried out at 800 rpm and 33 °C for 24 h in an Eppendorf Thermo mixer.

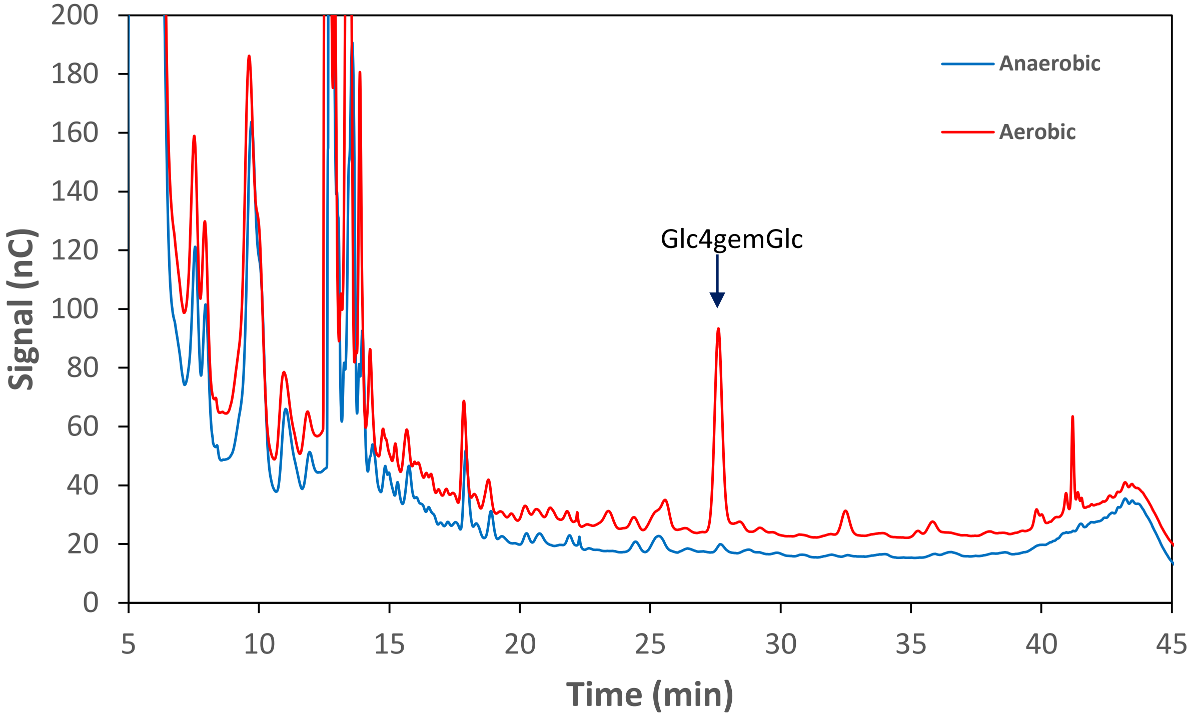

Figure S2: HPAEC-PAD profiles of products after saccharification of 10% steam exploded birch by Cellic™ CTEC2 under anaerobic (blue) and aerobic (red) conditions. Conditions were pH 5.0, 50 °C and 18 h of incubation. D-gluconic acid elutes after about 12 min where there is a lot of interfering peaks. Glc4gemGlc elutes much later around 28 h where there are no interfering peaks. It is readily seen that oxygen is required to produce Glc4gemGlc.
